# Intelligent Multi-Modeling Reveals Biological Mechanisms and Adaptive Phenotypes in Hair Sheep Lambs from a Semi-Arid Region

**DOI:** 10.3390/genes16070812

**Published:** 2025-07-11

**Authors:** Robson Mateus Freitas Silveira, Fábio Augusto Ribeiro, João Pedro dos Santos, Luiz Paulo Fávero, Luis Orlindo Tedeschi, Anderson Antonio Carvalho Alves, Danilo Augusto Sarti, Anaclaudia Alves Primo, Hélio Henrique Araújo Costa, Neila Lidiany Ribeiro, Amanda Felipe Reitenbach, Fabianno Cavalcante de Carvalho, Aline Vieira Landim

**Affiliations:** 1 Department of Animal Science, Luiz de Queiroz College of Agriculture (ESALQ), University of São Paulo (USP), Piracicaba 13418-900, SP, Brazil; 2 MBA in Data Science and Analytics, Luiz de Queiroz College of Agriculture (ESALQ), University of São Paulo (USP), 11 Pádua Dias Ave, Piracicaba 13418-900, SP, Brazil; fabioaugusto.rbr@gmail.com (F.A.R.); tjpsantos@outlook.com (J.P.d.S.); lpfavero@usp.br (L.P.F.); 3 School of Economics, Business and Accounting, University of São Paulo (USP), São Paulo 05508-900, SP, Brazil; 4 Department of Animal Science, Texas A&M University, College Station, TX 77843, USA; luis.tedeschi@tamu.edu; 5 Department of Animal and Dairy Science, University of Georgia, Athens, GA 30602, USA; alvesand@uga.edu; 6 Hamilton Institute, Department of Mathematics and Statistics, Maynooth University, County Kildare W23 F2H6, Ireland; danilo.stats@gmail.com; 7 Department of Animal Science, Vale do Acaraú State University (UVA), Sobral 62040-370, CE, Brazilhelioa.costa@gmail.com (H.H.A.C.); alinelandim@yahoo.com.br (A.V.L.); 8 Department of Animal Science, Federal University of Paraíba, Campus II, PB-079 Highway (km 12), Areia 58397-000, PB, Brazil; neilalr@hotmail.com; 9 Institute of Chemistry, University of Brasília, Brasília 70904-970, DF, Brazil

**Keywords:** advanced analytics, adaptation, phenotypic biomarker, meat traits, thermoregulatory responses

## Abstract

Background: Heat stress challenges small ruminants in semi-arid regions, requiring integrative multi-modeling approaches to identify adaptive thermotolerance traits. This study aimed to identify phenotypic biomarkers and explore the relationships between thermoregulatory responses and hematological, behavioral, morphometric, carcass, and meat traits in lambs. Methods: Twenty 4-month-old non-castrated male lambs, with an average body weight of 19.0 ± 5.11 kg, were evaluated under natural heat stress. Results: Thermoregulatory variables were significantly associated with non-carcass components (*p* = 0.002), carcass performance (*p* = 0.027), commercial meat cuts (*p* = 0.032), and morphometric measures (*p* = 0.029), with a trend for behavioral responses (*p* = 0.078). The main phenotypic traits related to thermoregulation included idleness duration, cold carcass weight, blood, liver, spleen, shank, chest circumference, and body length. Exploratory factor analysis reduced the significant indicators to seven latent domains: carcass traits, commercial meat cuts, non-carcass components, idleness and feeding behavior, and morphometric and thermoregulatory responses. Bayesian network modeling revealed interdependencies, showing carcass traits influenced by morphometric and thermoregulatory responses and non-carcass traits linked to ingestive behavior. Thermoregulatory variables were not associated with meat quality or hematological traits. Conclusions: These findings highlight the complex biological relationships underlying heat adaptation and emphasize the potential of combining phenomic data with computational tools to support genomic selection for climate-resilient and welfare-oriented breeding programs.

## 1. Introduction

Recent projections point to a 1.5 °C increase in Earth’s temperature by 2050 [1], which can largely affect animal production and biodiversity [2]. On the other hand, there is a growth in the planet’s population and, consequently, in the demand for food. These factors reinforce the need for studies to understand the impacts of climate change on animal thermoregulation and, specifically, the impact of heat stress on the productive performance of animals [3].

Adaptation to harsh environmental conditions is a complex process that involves the physiological, morphological, and behavioral responses of animals. These mechanisms can have genetic and non-genetic factors associated with them [4]. Several biological aspects are interconnected with thermoregulatory responses [5]. Most domestic animals can be classified as homeothermic beings, requiring the maintenance of a stable body temperature within adequate limits to ensure optimal physiological functioning. This thermal regulation is closely linked to responses in the animals’ behavior, such as reduced feeding, seeking shade, or modification in their activity in response to environmental variations [6].

It is also known that thermoregulation directly affects animal physiology, including hematological aspects, such as red and white blood cell counts, under conditions of heat stress. For instance, animals can present changes in blood concentrations (hemoconcentration) and reduced immunity, which can impact their health and performance [7]. Consequently, the carcass will have a lower weight, altering its entire body composition, such as reduced subcutaneous fat to dissipate excess heat, which will affect the quality of the meat, influencing characteristics such as marbling and texture [5].

Therefore, understanding and adequately managing animal thermoregulation are crucial to ensure their health, welfare, and productive performance. The application of advanced data analytics techniques can help analyze large datasets related to thermoregulation, behavior, hematology, carcass, and meat traits, allowing for a more comprehensive understanding of biological interactions within animal production systems [8]. This, in turn, can inform more effective and sustainable management decisions. Considering this perspective, intelligent multi-models are among the viable techniques for identifying more significant variables in an animal production system. These models are dedicated to understanding the intricate behavior of complex and dynamic systems or processes [9], which are valuable tools for unraveling the complexities inherent in the multivariable processes involved in animal adaptation [10].

Canonical correlation analysis (CCA) is a multivariate statistical technique used to understand and quantify the relationships between two sets of variables. It identifies linear combinations of the variables within each set (canonical variates) that are maximally correlated with each other [11,12]. MANOVA, which is also a multivariate statistical technique, allows for the simultaneous analysis of multiple dependent variables to determine whether there are significant differences between groups [13], being particularly useful in analyzing the impact of categorical independent variables on multiple continuous dependent variables simultaneously. With MANOVA, it is possible to evaluate how many factors, such as carcass performance metrics and commercial meat cuts, individually influence the thermoregulatory responses of animals, making it suitable for exploring the intricate dynamics of thermoregulatory responses in animals, combined with the canonical correlation analysis.

Given the large number of variables involved in adaptation to the thermal environment in the biological system, another efficient technique to reduce the number of variables is exploratory factor analysis [8]. The latent variables generated by this method could be used in a Bayesian network to represent the relationship between variables and capture probabilistic dependencies, generating insights based on graphs of joint multivariate probability distributions that represent conditional independence between variables [14]. Together, these four techniques can be used to understand relationships among variables, identify potential biomarkers, and explore complementary associations within the biological system.

The purpose of this study was to apply machine learning and multivariate statistical techniques to understand complex interactions between variables related to animal adaptation. By integrating these techniques, a more comprehensive understanding of the factors that influence thermoregulatory responses in animals will be revealed, especially in identifying phenotypic biomarkers. Therefore, the objectives of this study are as follows: (i) to identify the possible relationships between thermoregulatory responses with behavioral, morphological, blood, production, and carcass and meat traits in hair sheep lambs; and (ii) to reveal phenotypic biomarkers for adaptation to climate change through advanced analytical techniques as an auxiliary method.

## 2. Materials and Methods

### 2.1. Study Regulations, Location and Animals

All procedures and handling of experimental animals were conducted in accordance with protocols approved by the Ethics Committee on the Use of Animals of Vale do Acaraú State University—UVA (protocol n^o^. 003.04.015.UVA.504.02). The experiment was conducted at the Experimental Farm of Vale do Acaraú State University, in Sobral, CE, Brazil (3°41′09″ S; 40°20′58″ W; 75 m above sea level). During the experimental period, the average air temperature was 28 ± 4.3 °C and relative humidity was 70 ± 2.9%. The black globe temperature (BGT), which represents the thermal environment actually perceived by the animal—considering radiation, ambient temperature, and air movement—varied in the morning from 29 °C (at 08:00 h) to 32 °C (at 12:00 h) and in the afternoon from 33 °C (at 12:00 h) to 32 °C (at 17:00 h).

Twenty 4-month-old non-castrated male lambs, with an average body weight of 19.0 ± 5.11 kg, were used.

### 2.2. Herd Management

The animals were housed in 1.2 m^2^ individual stalls, equipped with feeders, drinkers, and salt blocks. The confinement period lasted 70 days, with 14 days for adaptation and 56 days for performance evaluation. During this period, the animals were fed a diet of Canarana erecta lisa grass (*Echinocloa pyramidalis*) and concentrated feed based on corn, soybean meal, and limestone, in a 60:40 roughage-to-concentrate ratio. The diet formulation followed the recommendations of the NRC [15] for the finishing category, with an estimated average weight gain of 150 g per day.

The diet was provided in two equal meals (8 a.m. and 4 p.m.), allowing for daily leftovers of approximately 10% of the total feed provided to allow voluntary intake. Water and mineralized salt were offered ad libitum. The animals were weighed weekly to estimate total weight gain (TWG, kg) and average daily weight gain (ADG, g = total weight gain/experimental period).

The components of the diet (grass and concentrate) were analyzed for dry matter (DM, method 934.01), ash (method 938.08), ether extract (EE, method 920.39), and crude protein (CP, method 934.13) following the AOAC [16] recommendations. Organic matter (OM) was calculated as the difference between DM and ash. Neutral detergent fiber (NDF) and acid detergent fiber (ADF) were analyzed according to Van Soest et al. [17]. Acid detergent lignin was determined using method 973.18D [16]. The methodology of Tilley and Terry [18] was employed for in vitro digestibility analyses of DM and OM (Table 1).

### 2.3. Data Collection

Thermoregulatory indicators (rectal temperature, respiratory rate, and heart rate) were measured twice daily—once in the morning (07:00–08:00 h) and once in the afternoon (13:00–14:00 h)—to capture variations associated with the natural thermal load in the semi-arid environment. At the end of the experimental period, blood samples were collected to assess the cumulative physiological effects of heat exposure. Morphometric measurements, ingestive behavior observations, and performance traits were also recorded at the end of the experiment, ensuring consistency in the evaluation of adaptive responses to heat stress.

#### 2.3.1. Thermoregulation

The thermoregulatory variables evaluated were respiratory rate (RR, breaths min^−1^), heart rate (HR, beats min^−1^), and rectal temperature (RT, °C). RR was measured by visually counting rib movements for 60 s. RT was recorded using a digital clinical thermometer inserted into the rectum. HR was obtained by auscultation with a stethoscope placed on the left thoracic region, counting heartbeats for 15 s and multiplying by four.

#### 2.3.2. Hematological Profile

Blood samples were collected from each animal by a licensed veterinarian via jugular venipuncture using vacuum tubes containing EDTA. The following hematological parameters were measured using an automatic hematology analyzer (Mindray BC-2800Vet, Shenzhen, China): red blood cell count (RBC; × 10^6^/mL), hemoglobin concentration (HC; g/dL^−1^), packed cell volume (PCV; %), mean corpuscular volume (MCV; fl), mean corpuscular hemoglobin (MCH; pg/hm), mean corpuscular hemoglobin concentration (MCHC; g/dL^−1^), segmented cells (SEG; cells/mL^−1^), lymphocyte (LYM; cells/mL^−1^), monocytes (MON; cells/mL^−1^), eosinophils (EOS; cells/mL^− 1^), platelets (PAL; cells/mL^−1^) and white blood cell (WBC, cells/mL^−1^).

#### 2.3.3. Behavioral Responses

The behavioral descriptors were recorded using the truncated focal sampling method [19]. This method was chosen because it allowed the animals to display their natural behavior, given their familiarity with handling practices, data collection, and handlers. Additionally, the animals were familiar with the professionals conducting the behavioral assessments. Each animal underwent observation in 10 min intervals, resulting in 6 observations per hour, 36 per day, and 108 per animal during the experimental period.

The behavioral categories were defined based on standardized ethological protocols. “Feeding” was characterized by active intake of feed, identified by head-down movements toward the feeder and jaw activity. “Rumination” was defined as the regurgitation and re-chewing of cud, typically identified by rhythmic jaw movements while the head remained elevated or in a neutral position. “Idleness” was defined as the absence of both feeding and rumination, with the animal standing or lying quietly, not engaged in locomotion or social interaction. Observers were trained to ensure consistency and reliability in identifying and recording these behaviors throughout the trial.

#### 2.3.4. Non-Carcass Components and Carcass Performance

Prior to slaughter procedures, the animals underwent 16 h solid food fasting. After fasting, they were weighed, and the fastened carcass weight at slaughter (FCW) was obtained. Slaughter was conducted following the regulations of industrial inspection and sanitation of animal products [20]. After evisceration, non-carcass constituents were measured (blood volume, lung weight, heart weight, kidney weight, liver weight, and spleen weight). The hot carcass was weighed (HCW) and stored in a cold room at a temperature of 4 °C for 24 h; finally, the cold carcass was weighed (CCW).

#### 2.3.5. Morphometric Measurements

The morphometric measurements of the carcass were performed using a measuring tape graduated in centimeters, as described by Cesar and Sousa [21] and Osório et al. [22]. Body length (BL), thorax perimeter (TP), shank circumference (SHANKCIRC), and the loin eye area (LEA) of the *longissimus lumborum* muscle exposed on the 12th thoracic rib were measured using the geometric method [23].

#### 2.3.6. Commercial Meat Cuts

To obtain the meat cuts and carcass composition, the specimens were longitudinally sectioned using a butcher band saw. The commercial meat cuts of interest for this study, namely shank, rib, and loin, were extracted from the left-half carcasses.

#### 2.3.7. Meat Traits

Carcass conformation, fat cover, and perirenal fat were assessed visually, following the procedure outlined by Cezar and Sousa [24]. Conformation was scored on a scale of 1 (poor) to 5 (excellent), based on the development of muscle and fat tissue in relation to the skeletal structure. Fat finishing was evaluated on the same 1 to 5 scale, where 1 indicated absence and 5 represented excessive coverage, considering the quantity and distribution of subcutaneous fat in the chilled carcass.

Marbling was visually assessed based on the amount of intramuscular fat present in the muscle, using a 5-point scale, as described by Osório et al. [25], where 1 = absent and 5 = excessive. Texture was also evaluated using a 5-point scale, where 1 = very coarse and 5 = very fine.

#### 2.3.8. Meat Physical Characteristics

The pH assessment was conducted using a portable pH meter equipped with an electrode tailored for semi-solid substances. That instrument possesses a measurement span from 0 to 14. During the evaluation process, the electrode’s tip was inserted perpendicularly into the muscle mass at three distinct spots, allowing time for stabilization. The resultant data were meticulously logged into a table, categorizing the samples by establishment and noting the time of each collection.

The temperature measurements of the meat cuts were carried out using a handheld probe thermometer, inserted perpendicularly into the muscle mass at three different points to a depth of two centimeters.

### 2.4. Statistical Methods

Initially, descriptive statistics of all variables involved in this study were explored, which are represented in Table 2.

#### 2.4.1. Canonical Correlation Analysis (CCA)

Canonical correlation analysis (CCA) was used to determine the correlation coefficient (r_c_), i.e., to estimate the magnitude of correlations between thermoregulatory, productive, and morphometric responses, as well as non-carcass components. This technique is based on the correlation between a linear combination of a set of dependent variables ($X_{p}$) and a linear combination of another set of independent variables ${(W}_{q})$. Linear combinations of variables are very useful for comparison and prediction [26]. These linear combinations of sets of variables can be defined as follows:

$$U_{i}=a_{i1}X_{1}+a_{i2}X_{2}+\cdots a_{ip}X_{p}$$

$$V_{i}=b_{i1}W_{1}+b_{i2}W_{2}+\cdots b_{iq}W_{q}$$

where:

$a_{ip}$ and $b_{iq}$ are canonical coefficients;

$U_{i}$ and $V_{i}$ are the *i*th pair of canonical variables.

$U_{1}$ and $V_{1}$ form the first pair of canonical variates, which is associated with the first canonical correlation, as follows:

$$r_{1}=\frac{Côv\left(U_{1},V_{1}\right)}{\sqrt{{Vâr(U}_{1})Vâr(V_{1})}}$$

The percentage of variance explained by the first canonical variates is $U_{Xi}^{2}$ and $V_{Wi}^{2}$:

$$U_{Xi}^{2}=\frac{\sum_{j=1}^{p}a_{ij}^{2}}{p}and\;V_{Wi}^{2}=\frac{\sum_{j=1}^{q}b_{ij}^{2}}{q}$$

in which:

*p* is number of variables of *X*;

*q* is number of variables of *W*.

The total number of pairs of canonical variates is defined by the minimum value between *p* and *q*.

Finally, the CCA produces a set of new variables called the canonical functions, which are linear combinations of the original variables, as reported in the following equation:

$$C_{F}=d_{1}X_{1}+d_{2}X_{2}++d_{3}X_{3}+\ldots d_{n}X_{n}$$

in which:

$d_{n}$ is the canonical coefficients, which indicate the contribution of each variable in the composition of *C_F_*;

X is the scores of the n original variables.

Initially, the assumption of multivariate normality was verified through the Mardia test [27,28], followed by the multicollinearity diagnosis for variables within the same group using residual correlations through multivariate analysis of variance [29]. Morphological response, productive variables, hematological profile and ingestive behavior were considered dependent, while thermoregulatory responses were considered independent. Prior to the application of multivariate analyses, Pearson’s correlation coefficients were calculated among the independent variables to assess potential collinearity and ensure the independence of predictors. Only the first canonical function was considered. A significance level of 0.05 was adopted. Finally, the square correlation coefficient was calculated and represented the amount of variation an ideally weighted canonical variable is explained by another ideally weighted canonical variable [30].

#### 2.4.2. MANOVA

Multivariate analysis of variance (MANOVA) is a statistical analysis method used to compare mean vectors of more than one dependent variable across one or more groups. Unlike ANOVA, which analyzes the difference in means among groups for a single dependent variable, MANOVA considers multiple dependent variables simultaneously. This approach helps to explain the complex interdependencies and effects among variables, assessing whether the means of certain groups are different in a multidimensional space defined by those variables. MANOVA is particularly useful when dependent variables are correlated, allowing for a more nuanced understanding of the relationships among them.

MANOVA relies on several key assumptions, including the independence of observations, multivariate normality of dependent variables, and homogeneity of variance–covariance matrices across groups. These assumptions are crucial for the validity of the method. Different test statistics, such as Pillai’s trace, Wilks’ lambda, Hotelling’s trace, and Roy’s largest root, are used in MANOVA to determine the significance of observed differences among group means.

*Analysis of variance*

*Y*_1_ = *X*_1_ + *X*_2_ + *X*_3_ + … + *X_n_*

   (metric)                       (nom-metric)           

*Multivariate analysis of variance*

MANOVA was performed to identify the main biomarkers of animal adaptation according to indicators in groups

*Y*_1_ + *Y*_2_ + *Y*_3_+ … + *Y*_n_ = *X*_1_ + *X*_2_ + *X*_3_ + … + *X_n_*

(metric)                        (non-metric)

Thus, in the univariate approach, the null hypothesis evaluates the statistical significance of the means of the dependent variable among n groups, given by:

$$H_{0}=\mu_{1}=\mu_{2}=\ldots {=\mu }_{n}$$

$$H_{0}=\left(\begin{array}{c} \begin{array}{c} \mu_{11}\\ \mu_{21} \end{array}\\ \vdots \\ \begin{array}{c} \vdots \\ \mu_{p1} \end{array} \end{array}\right)=\left(\begin{array}{c} \begin{array}{c} \mu_{12}\\ \mu_{22} \end{array}\\ \vdots \\ \begin{array}{c} \vdots \\ \mu_{p2} \end{array} \end{array}\right)=\ldots =\left(\begin{array}{c} \begin{array}{c} \mu_{1n}\\ \mu_{2n} \end{array}\\ \vdots \\ \begin{array}{c} \vdots \\ \mu_{pn} \end{array} \end{array}\right)$$

That is, the null hypothesis, that all groups have equal mean vectors, as opposed to the alternative hypothesis that at least two groups have different mean vectors, is tested.

To evaluate the association between thermoregulatory responses and multiple phenotypic traits, a series of MANOVA tests was conducted. The dependent variables were the three thermoregulatory indicators: rectal temperature (RT), respiratory rate (RR), and heart rate (HR). Each MANOVA model tested a biologically coherent group of independent variables, including behavioral responses, carcass traits, non-carcass components, commercial meat cuts, and morphometric traits, jointly against this same vector of thermoregulatory outcomes.

Prior to applying MANOVA, key assumptions were assessed. Multivariate normality was tested using the Mardia test, and homogeneity of covariance matrices was evaluated via Box’s M test. To minimize collinearity within groups, variables were pre-screened using pairwise correlation matrices, and highly correlated indicators (r > 0.85) were excluded or combined.

As multiple MANOVA tests were performed independently across different groups of predictors, a Benjamini–Hochberg False Discovery Rate (FDR) correction was implemented to control the expected proportion of false positives. Both raw and adjusted *p*-values are reported in the results to provide transparency and account for multiple testing.

This approach allowed for the identification of phenotypic domains significantly associated with thermoregulatory outcomes while ensuring statistical rigor and interpretability across multiple biological systems.

#### 2.4.3. Exploratory Factor Analysis

The exploratory factor analysis was carried out only with the indicators that the canonical analysis showed significance with the thermoregulatory responses. To assess the suitability of the data for factor analysis, Bartlett’s test of sphericity (*p* < 0.001) and the Kaiser–Meyer–Olkin (KMO) measure of sampling adequacy (KMO ≥ 0.50) were performed, confirming the adequacy of the data structure for EFA. Orthogonal rotation was used. The objective of factor analysis is to reduce the number of variables (phenotypes) into latent variables. The factor analysis model is expressed by Equation (1):

(1) $$X_{1}=a_{11}\times F_{1}+a_{12}\times F_{2}+\ldots +a_{1m}\times F_{m}+e_{p}\;X_{2}=a_{21}\times F_{2}+a_{21}\times F_{2}+\ldots +a_{2m}\times F_{m}+e_{p}\;\vdots \;X_{p}=a_{p1}\times F_{1}+a_{p1}\times F_{2}+\ldots +a_{pm}\times F_{m}+e_{p}$$

where $X_{p}$ is the pth score of the standardized variable (*p* = 1, 2, …, *m*), $F_{m}$ is the extracted factor, $a_{pm}$ is the factor loading, and $e_{p}$ is the error.

Factor scores for each group were estimated by multiplying standardized variables by the coefficient of the corresponding factor score, as follows in Equation (2)

(2) $$F_{1}=d_{11}\times X_{1}+d_{12}\times X_{2}+\ldots +d_{1j}\times X_{jp}\;F_{2}=d_{21}\times X_{2}+d_{21}\times X_{2}+\ldots +d_{2j}\times X_{jp}\;\vdots \;F_{j}=d_{p1}\times X_{1}+a_{j1}\times X_{2}+\ldots +d_{jp}\times X_{jp}$$

where: $F_{j}$ is the jth factor extracted, $d_{pj}$ is the factor score coefficient, and *p* is the number of variables [31].

#### 2.4.4. Bayesian Network Analysis

Bayesian analysis was performed to reveal the complex relationships among the variables under study. To reduce model complexity and improve interpretability, the latent variables extracted were used and renamed from the exploratory factor analysis (EFA) step. These composite indicators represent multidimensional phenotypic traits related to thermoregulation, behavior, morphometry, and performance.

The Bayesian network (BN) was constructed using the Hill-Climbing (HC) structure-learning algorithm implemented in R (version 4.4.3) via the *bnlearn* package. The scoring criterion applied was the Bayesian Information Criterion (BIC), which balances model fit and complexity. To ensure the stability and robustness of the inferred structure, a bootstrap resampling procedure with 1000 iterations was conducted. Edges retained in the final network were those present in more than 70% of the resampled networks, representing consistent and reliable conditional dependencies.

In a Bayesian Network, a directed acyclic graph represents probabilistic relationships between random variables. Each node corresponds to a variable, and the edges indicate conditional dependencies. The absence of an edge implies conditional independence, given the variables directly connected. This structure allows for the identification of both direct and indirect effects among variables, enhancing biological interpretability. Formally, the BN is represented as $BN=(G,X_{V})$, where *G* is an acyclic graph composed of nodes V and edges E, describing the probabilistic relationships among the variables in the vector $X_{V}$ = ($X_{1\ldots .}X_{k})$, with *k* representing the number of random variables involved [32].

## 3. Results

### 3.1. Identifying the Relationship of Variables with Climatic Adaptation

A summary of the canonical correlation analysis for the nine types of indicators (sets of independent variables) with the thermoregulatory responses of sheep in the Brazilian semi-arid region is shown in Table 3. Only behavioral responses (*p* = 0.078), non-carcass components (*p* = 0.002), carcass characteristics (*p* = 0.027), commercial meat cuts (*p* = 0.032), and morphological responses (*p* = 0.029) influenced thermoregulatory responses. The opposite was observed for red blood cells (*p* = 0.520), white blood cells (*p* = 0.669), meat physical characteristics (*p* = 0.919), and meat traits (*p* = 0.437). Therefore, the results presented below are only for standardized canonical coefficients of the significant canonical correlations.

#### 3.1.1. Behavioral Responses

**Figure 1:**
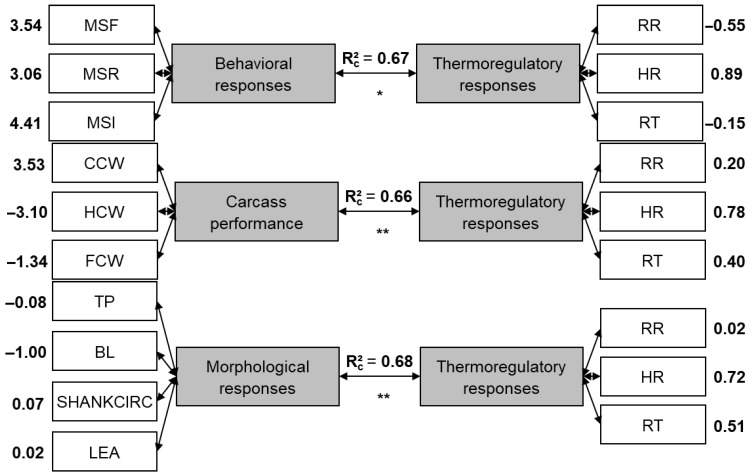

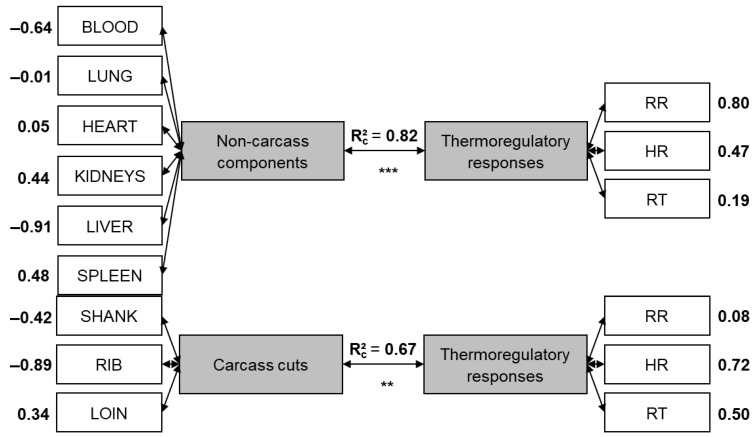
Canonical standardized coefficients of thermoregulation with behavioral response, carcass performance, non-carcass components, commercial meat cuts and morphometric responses. RR: respiration rate; HR: heart rate; RT: rectal temperature; MSF: minutes spent feeding; MSI: minutes spent in idleness; MSR: minutes spent ruminating, CCW: cold carcass weight; FCW: fasting carcass weight; HCW: hot carcass weight, BLOOD: blood volume; LUNG: lung weight; HEART: heart weight; KIDNEYS: kidney weight; LIVER: liver weight; SPLEEN: spleen weight; SHANK: shank weight; TP: thorax perimeter; BL: body length; SHANKCIR: shank circumference; LEA: loin eye area. Note: * *p* < 0.05 and ** *p* < 0.001, *** *p* < 0.0001.

The correlation between behavioral and thermoregulatory responses measured by the canonical standardized coefficients is shown in Figure 1. It was observed that the longer the lambs are feeding (3.54), ruminating (3.06), or remaining idle (4.41), the higher their heart rate (0.89), which suggests that these activities may have a stimulating effect on the sheep’s cardiovascular system. It was also found that the more minutes they spent on feeding, rumination, and idling (mainly), the lower the respiratory rate (−0.55), which may indicate that an increase in RR is associated with more active behavior or with agitated sheep. Finally, the rectal temperature had a small canonical correlation with behavioral responses (−0.15), which suggests that variations in rectal temperature are not strongly associated with the behavioral activities observed in sheep.

#### 3.1.2. Carcass Traits

The correlation between thermoregulatory responses and carcass performance is shown in Figure 1. Heart rate (0.78) and rectal temperature (0.40) showed a negative correlation with fasting carcass weight (−1.34), which implies that higher heart rate and rectal temperature are associated with smaller sheep and lighter fasten carcasses. Furthermore, the respiration rate (0.20) showed a negative but smaller correlation with fasting carcass weight, which suggests a trend that higher respiration rates may be associated with smaller sheep. Additionally, the cold carcass weight (3.53) demonstrated a positive correlation with thermoregulatory responses; i.e., the heavier the cold carcass weight, the higher the heart rate and rectal temperature, while hot carcass weight (−3.10) showed a negative correlation, which indicates that heavier hot carcass weights are associated with lower thermoregulatory responses, aligned with the fasting carcass weight results.

#### 3.1.3. Morphological Responses

Body length (−1.00) had a negative correlation with heart rate and rectal temperature, which suggests that shorter body length is associated with lower heart rates and rectal temperatures. On the other hand, the other variables involved did not seem to have a significant correlation, which includes thorax perimeter (−0.08), shank circumference (0.07), and loin eye area (0.02) with thermoregulatory variables, or respiratory rate with morphological responses variables; that is, these morphological characteristics have limited impact on thermoregulatory responses, or vice versa, and the same occurs for respiratory rate with morphological variables.

#### 3.1.4. Non-Carcass Components

Kidneys (0.44) and spleen (0.48) showed a positive correlation with respiratory rate (0.80) and heart rate (0.47), which implies that these organs may play a role in the thermoregulatory system or have their functioning relevantly affected by it. On the other hand, the blood (−0.64) and liver (−0.91) showed a negative correlation, which suggests that higher levels of blood and liver components are associated with decreased thermoregulatory responses (potentially more efficient), while lung (−0.01) and heart (0.05) did not demonstrate a strong correlation with thermoregulatory responses, which implies that these organs may have minimal influence on the regulation of body temperature, or vice versa. Finally, the rectal temperature (0.19) showed a low correlation with non-carcass components, which suggests that variations in rectal temperature may not strongly correlate with changes in non-carcass components.

#### 3.1.5. Commercial Meat Cuts

Rib (−0.89) and shank (−0.42) showed a negative correlation with heart rate (0.72) and rectal temperature (0.50), which suggests that heavier rib and shank cuts are associated with lower heart rates and rectal temperatures. Furthermore, the respiration rate demonstrated very little correlation (0.08), which suggests that respiration rate has minimal influence or is minimally influenced by the weight of meat cuts. Finally, the loin (0.34) showed a small positive correlation with the thermoregulatory responses, which implies a trend that heavier loin cuts are associated with higher heart rates and rectal temperatures.

### 3.2. Identifying Phenotypic Biomarkers of Adaptation

Based on the results of canonical correlation analysis, the MANOVA algorithm was used to confirm the significance for the correlations found between the independent variables (behavioral responses, non-carcass components, carcass performance, commercial meat cuts, and morphological responses; Table 4) with thermoregulatory responses. The main phenotypic markers related to thermoregulatory responses were minutes spent in idleness (*p* = 0.042), cold carcass weight (*p* = 0.005), blood (*p* = 0.004), liver (*p* = 0.017), spleen (*p* = 0.016), shank (*p* = 0.004), chest circumference (*p* = 0.011) and body length (*p* = 0.018). In addition, lung (*p* = 0.070) showed a trend towards significance, suggesting a potential effect that encourages further investigation with a larger sample size or additional data.

### 3.3. Understanding the Correlation of the Biological System

**Figure 2:**
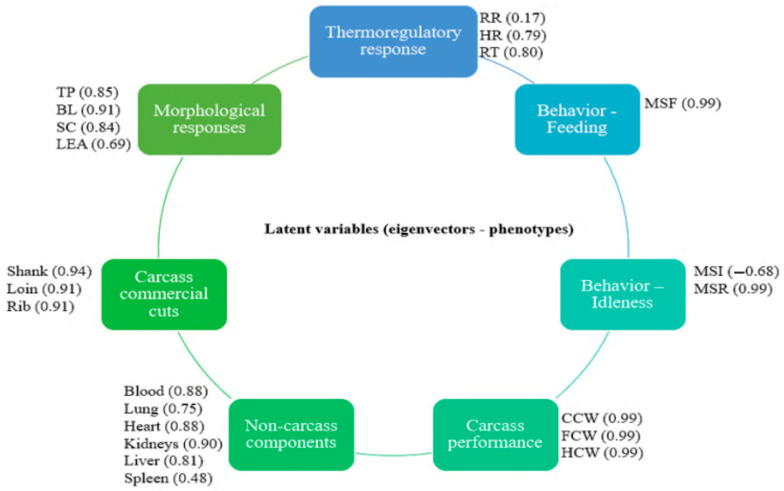
Renamed latent variables and eigenvalues of the original variables according to exploratory factor analysis.

**Figure 3:**
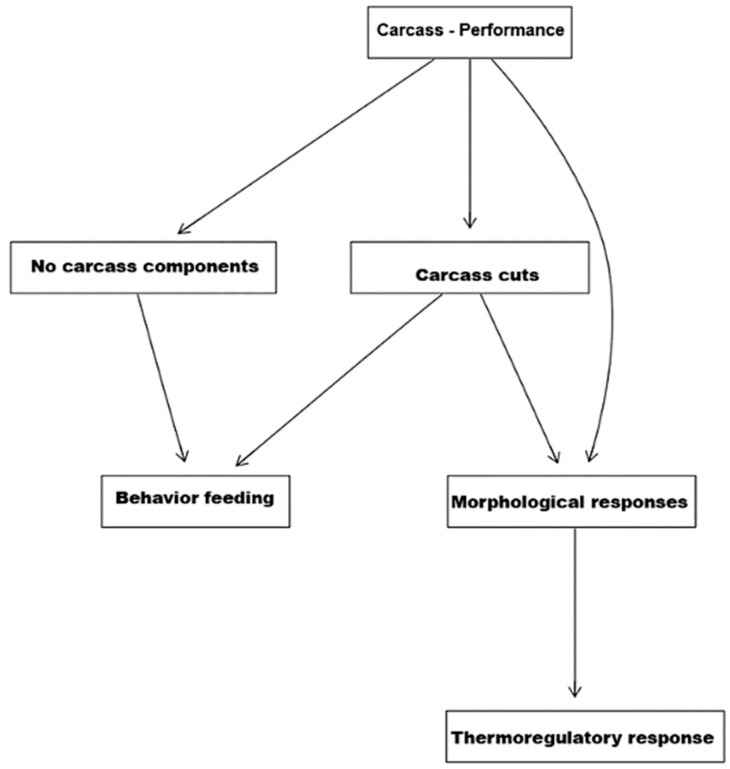
Bayesian network between latent variables showing the correlation between behavioral, thermoregulatory, productive and morphometric responses of hair sheep in semi-arid area of Brazil.

Of the six indicators that showed a significant correlation with the thermoregulatory responses indicated in the canonical correlation analysis, the exploratory factor analysis grouped them into seven latent variables (Figure 2): carcass traits, commercial meat cuts, non-carcass components, behavior response—idleness, behavior response—feeding, morphometric responses, and thermoregulatory responses. The correlation between these variables is presented in Figure 3. The Bayesian network analysis showed that these variables are correlated with direct and indirect effects, except for idleness behavior; when specifying such a relationship, it was observed that carcass performance directly influenced commercial meat cuts and non-carcass components, which, in turn, are influenced by the animal’s ingestive behavior. Still, carcass performance was influenced by morphometric responses. Finally, thermoregulatory responses were conditionally independent from all the other latent traits given the morphometric measures.

## 4. Discussion

This study presented a comprehensive analysis of the intricate relationship between thermoregulatory responses and behavioral, productive, morphometric responses and carcass and meat traits of sheep from the Brazilian semi-arid region. Using mechanistic models, significant insights were obtained into the factors that influence thermoregulation and general physiological functioning in these animals. Notably, behavioral responses, non-carcass components, carcass performance, commercial meat cuts, and morphological responses emerged as key variables that can be considered phenotypic biomarkers for thermoregulation or adaptation to climate change. This suggests that a holistic understanding of animal production systems, encompassing multiple biological and environmental factors, is crucial to optimizing management practices to ensure animal health, welfare, and productivity.

These findings highlight the importance of incorporating advanced analytical techniques into animal production research to reveal complex interactions between variables. By identifying significant relationships between behavioral, physiological, and morphological factors, as well as thermoregulatory responses, it is possible to adapt management strategies to mitigate the impacts of environmental stressors, such as heat, on animal performance. Furthermore, this study underscores the value of interdisciplinary approaches in addressing pressing challenges in agriculture, emphasizing the need for collaboration between animal scientists, statisticians, and computing experts to leverage data-driven insights for sustainable livestock production.

In essence, this discussion unveils the intricate interplay between behavioral, physiological, and morphological factors in shaping the multifaceted nature of thermoregulatory responses in sheep from a semi-arid region. By elucidating the complex dynamics at play, researchers are better equipped to devise targeted strategies aimed at bolstering resilience and fostering adaptation in response to the ever-changing environmental conditions characteristic of semi-arid regions. This holistic perspective underscores the importance of considering diverse factors in optimizing sheep welfare and productivity, ultimately contributing to sustainable agricultural practices in challenging environments.

The canonical correlation and MANOVA analysis provided valuable insights into the complex interplay between thermoregulatory responses, behavioral responses, carcass characteristics, and the other variables analyzed in Brazilian semi-arid sheep. Through both analysis methods, it was determined that several variables showed significance, shedding light on their potential roles in influencing thermoregulation and overall physiological functioning. Notably, behavioral responses, non-carcass components, carcass performance, commercial meat cuts, and morphological responses showed a significant correlation with thermoregulatory responses in both canonical correlation analysis and MANOVA.

Regarding the thermoregulatory variables, heart rate proved to be the most affected, presenting a significant relationship with all the groups of independent variables mentioned above; as for rectal temperature, there was a significant correlation with carcass performance, commercial meat cuts, and morphological responses, while for respiratory rate, there was a significant correlation with behavioral responses and non-carcass components.

When analyzing the relationship between thermoregulation and behavioral responses, minutes spent in idleness showed a positive correlation with heart rate and a negative correlation with the respiratory rate of sheep; i.e., the more time sheep spend idling, the higher their heart rate tends to be, while their respiration rate tends to decrease. This correlation reflects a complex physiological response. Elevated heart rates are commonly indicative of heat stress in sheep, prompting increased muscular activity. This heightened activity serves to regulate their respiratory rate and enhance vascularization, facilitating heat dissipation through the skin, as explained by Meneses et al. [33]. Consequently, as sheep remain idle for extended periods, their body temperature tends to decrease gradually, leading to a corresponding reduction in their respiratory rate. This series of physiological responses underscores the adaptability and resilience of sheep to environmental challenges.

In the context of the relationship between non-carcass components and thermoregulatory variables, blood volume and liver weight showed a negative correlation with both respiratory and heart rate; that is, the higher or heavier these are, the lower the respiratory and heart rates tend to be. Given that vascularization can be a key factor in heat dissipation in sheep [34], it is reasonable to infer that sheep with a higher blood volume may be more efficient in thermoregulation. The presence of a larger volume of circulating blood allows for a broader distribution through peripheral blood vessels, which, in turn, facilitates thermal exchange with the environment. Therefore, the increase in blood volume may allow the sheep to maintain adequate body temperatures.

When looking at the correlations between variables related to carcass performance, cold carcass weight demonstrated a positive correlation with heart rate and rectal temperature; that is, sheep with heavier cold carcass weight tend to have higher heart rates and rectal temperatures, which may be explained by the metabolic demand associated with increased muscle and metabolic development, as concluded by Seixas et al. [35], who compared two hair breeds of sheep (Morada Nova and Santa Inês) and found that smaller animals were more heat-tolerant, also in line with [36], who inferred that the smaller body size is an adaptation to warmer climates since body size affects energy requisites for maintenance, growth and production [37].

These findings regarding body size and thermoregulation must be interpreted with caution, as the literature offers divergent perspectives. On one hand, smaller body size is generally considered advantageous in hot climates due to a higher surface area-to-volume ratio, which facilitates heat loss and reduces metabolic heat production [36,37]. On the other hand, the observed negative associations between body length and thermoregulatory indicators (i.e., heart rate and rectal temperature) in our study suggest that longer-bodied animals may exhibit more efficient heat dissipation, possibly due to greater vascular surface area or limb conformation [38,39]. These findings are not necessarily contradictory but rather reflect different physiological and morphological strategies for coping with thermal stress. The balance between metabolic load and heat exchange capacity may vary depending on body conformation, breed characteristics, and adaptive history.

In the analysis of the correlations between commercial meat cuts and morphological responses, shank weight showed a negative correlation with heart rate and rectal temperature; i.e., the heavier the sheep shanks, the lower the heart rate and rectal temperature tend to be. Shank weight may reflect the body size and composition of sheep, including muscle and fat content. An increase in shank weight may be associated with greater efficiency in thermoregulation, resulting in lower heart rate and rectal temperature. Body length had a negative relation with heart rate and rectal temperature; that is, the larger the body of the sheep, the lower its heart rate and rectal temperature tend to be. Therefore, an increase in body length may indicate a trend to a more efficient thermoregulatory system with lower heart rates and rectal temperatures. Although these last two conclusions are contrary to those pointed out above on the correlation between smaller bodies and thermoregulation efficiency in sheep, there are also lines of studies, such as the one developed by Silanikove [38], that pointed out that larger animals have a lower metabolic rate and so, again, heat at a slower rate. Additionally, it is also seen that animals with long, thin appendages reduce heat gain and increase heat loss [39]. Therefore, this corroborates and possibly also confirms the causality between longer bodies with larger shanks and greater efficiency in the thermoregulatory system of sheep, resulting in lower heart rates and rectal temperatures.

The identification of phenotypic biomarkers with different responses of the biological system demonstrates that the science of thermal biology is multidisciplinary, and there is a need for more studies focused on behavior, non-carcass components, morphometry, and not just productive characteristics like most studies published in animal science, especially in the dairy cattle production chain [40,41,42].

The relationship between behavioral and thermoregulatory responses is already widely discussed, as it is an animal’s response to seeking ways to dissipate heat, such as looking for shade or even reducing the consumption of dry matter to reduce the production of metabolic heat [43]. However, few studies have related ingestive behavior to thermoregulatory responses in small ruminants. The importance of minutes spent in idleness shows that it is an important behavior for the thermal balance, as the animal is not consuming feed, in addition to being an indication of animal welfare, as the animal is resting. Further inference cannot be made, but more specific studies are encouraged to understand the relationship between ingestive behavior, dry matter intake and thermoregulatory response in production animals.

The identification of cold carcass weight as an adaptive biomarker shows the importance of ensuring a comfortable thermal environment for the animals, as it directly impacts the farm’s economic indicators. The relationship between thermoregulatory responses and productive responses in bulls has already been reported by Silveira et al. [12] using canonical correlation analysis. It is also worth mentioning that the animals in this study were raised in a semi-arid region with average temperatures of 28 °C.

The identification of blood as the main biomarker within non-carcass components is justified because it acts as a heat transport medium, facilitating heat transfer between internal organs and the skin, adjusting blood flow to regulate heat dissipation or conservation, interacting with neural and hormonal feedback systems to maintain stable body temperature [44]. Other studies carried out with sheep have already demonstrated the importance of blood volume as a predictive variable related to the adaptation of animals subjected to high temperatures [10].

Similar to blood volume, a heavier liver is beneficial because this organ also functions in thermoregulation due to its high level of metabolic activity, constant heat production, and ability to influence the redistribution of heat through blood flow [45]. It is also worth mentioning that in response to signals from the central nervous system, the liver regulates (increasing or reducing) the rate of metabolic activity to adjust heat production. For example, during heat exposure, the liver may increase its metabolic activity to produce less heat and help maintain body temperature.

The carcass morphometric variables that were identified as phenotypic biomarkers of adaptation rectify their relationship with the morphometric variables, which corroborates studies by Silveira et al. [10] and Mcmanus [34,46]. This correlation is not new, as has already been discussed on the previous topic.

Each of the phenotypic markers identified in this study contributes to thermoregulation through distinct biological mechanisms. For instance, increased blood volume facilitates peripheral perfusion and enhances heat dissipation via convection and radiation [44]. Liver and spleen weights reflect metabolic and immunological demands, which are directly linked to endogenous heat production and circulatory dynamics [45]. Cold carcass weight may relate to metabolic rate and tissue insulation capacity, influencing heat retention. Chest circumference and body length contribute to the body’s surface area-to-volume ratio, which affects the efficiency of heat exchange with the environment. Lastly, behavioral parameters such as idleness can be seen as passive thermoregulatory strategies aimed at minimizing heat production under environmental stress [4]. Together, these findings support the relevance of integrating behavioral, physiological, and morphological data to identify reliable phenotypic biomarkers for heat tolerance in small ruminant animals [47,48].

It is noteworthy that red and white blood cell counts, as well as meat quality traits (such as pH, texture, and marbling), were not significantly associated with thermoregulatory measures in this study. While this may partly reflect the limited statistical power due to sample size, it may also suggest a true physiological decoupling between acute thermoregulatory responses and certain systemic or post-mortem characteristics. Blood cell counts, for instance, are often tightly regulated homeostatically and have been proposed as thermal stress biomarkers in goats [10], dairy cows [47], and beef cattle [49]. However, the absence of significant associations in this study may be related to breed-specific thermal adaptation mechanisms. Regarding meat quality traits, these are influenced by several post-mortem processes and metabolic factors that are not necessarily linked to short-term thermoregulatory mechanisms. These findings suggest that although thermoregulation is closely related to hematology, behavior, and morphometric responses, its relationship with specific blood parameters and meat quality traits may be more complex or indirect. Future studies with larger sample sizes and breed comparisons are needed to clarify these associations, particularly in thermotolerant animals.

By integrating factor analysis and Bayesian network approaches, it was possible to streamline the interrelationships between thermoregulatory, behavioral, morphometric, and productive responses. Factor analysis was used to reduce the initial set of 22 variables to 7 latent variables. These latent variables represent more direct biological meanings than phenotypic traits [14,32]. Subsequently, the Bayesian network revealed the direct and indirect interrelationship of these variables through seven connections, which was not possible to clarify through canonical correlation analysis and MANOVA.

Exploring these relationships, it can be seen that thermoregulatory responses do not directly impact productive responses, as many studies suggest. What the Bayesian network reveals is that morphometric responses have an impact on thermoregulatory responses, which, in turn, have an impact on productive performance. This makes sense since it is known that animals under heat stress reduce their feed intake, which impacts their growth and performance [4,12], particularly those subjected to chronic heat stress or high temperatures such as the animals in this study. This assumption is rectified when the eating behavior directly related to the sizes of commercial meat cuts and non-carcass components is analyzed; that is, eating behavior must be directly related to the size of the carcass, which influences the animal’s productive responses. Finally, the idleness behavior was not related to any variable, possibly because it is analogous to the feeding behavior.

## 5. Limitations

One of the main limitations of this study is the relatively small number of animals (*n* = 20), which may limit the generalizability of the results to larger populations. However, it is important to emphasize that the primary aim of this research was to apply and evaluate an integrative multi-modeling strategy capable of revealing complex biological correlations underlying thermoregulatory adaptation in hair lambs raised under semi-arid conditions. Despite the limited sample size, this study effectively combined multivariate statistical techniques, including canonical correlation analysis, exploratory factor analysis, and Bayesian networks, to extract structured information from a high-dimensional phenotypic dataset.

This modeling framework represents a methodological advance in animal phenomics, providing an alternative to traditional univariate methods, which often fail to capture the non-linear and multi-layered nature of adaptive biological processes [50]. Moreover, the proposed approach holds interdisciplinary potential and may be applied in other biological systems, such as plant responses to environmental stressors, where phenotypic complexity also plays a crucial role.

Finally, it is important to note that the statistical methods used in this study (e.g., canonical correlation and Bayesian networks) are designed to identify associations rather than causal relationships. Therefore, the biological interpretations presented should be understood as indicative patterns, serving as a foundation for hypothesis generation rather than conclusive evidence of causality [47].

## 6. Conclusions

Thermoregulatory responses are related to behavioral and productive responses, non-carcass components, and non-commercial meat cuts but not with the physical traits of meat and red and white blood cells. In addition, minutes spent in idleness, cold carcass weight, blood volume, liver and spleen weights, shank size, chest circumference, and body length are the main phenotypic biomarkers of climate adaptation. Finally, morphometric responses influence thermoregulatory responses, commercial meat cuts and non-carcass components. These last two are directly related to the ingestive feeding behavior of animals.

The use of intelligent methodologies is recommended in studies to investigate and elucidate the underlying mechanisms that drive the correlations observed between thermoregulation and many physiological and morphological parameters in production animals, particularly the phenotypic biomarkers found, due to the importance they have demonstrated in this context of welfare and animal adaptation. Furthermore, longitudinal studies that track changes in thermoregulatory responses over time, and in different environmental conditions, could provide valuable information about the adaptive capacity of livestock populations to climate change.

These results underscore the importance of integrative approaches combining phenomics and computational modeling as a basis for future genomic selection strategies in breeding programs focused on climate resilience and animal welfare.

## Figures and Tables

**Table 1:** Composition of the dietary components.

Nutrients (%)		Dietary Components	
	Grass	Concentrated	Diet
Dry Matter (DM)	91.8	92.9	92.2
Organic Matter (OM)	87.2	94.5	90.1
Crude Protein	5.16	36.8	17.8
Ether Extract	1.28	4.00	2.37
Neutral Detergent Fiber	75.8	16.9	52.2
Acid Detergent Fiber	43.0	9.60	29.6
Hemicellulose	32.5	7.30	22.4
Cellulose	34.7	9.43	24.6
Lignin	4.61	0.37	2.91
In vitro DM digestibility	31.0	73.0	47.8
In vitro OM digestibility	38.7	81.3	55.7

Canarana (*Echinocloa pyramidalis*); concentrated (corn = 39.7%; soybean meal = 59.9% and limestone = 0.40%).

**Table 2:** Descriptive statistics of the variables under study.

Group	Variable	Unit	Min	Mean	Median	SD	IQR	Max
Thermoregulatory responses	RR	breaths min^−1^	84.5	105.58	107.50	9.53	9	125
	HR	bpm	36	68.13	66.50	19.62	14.75	111
	RT	°C	38.95	39.5	39.55	0.22	0.17	39.8
Behavioral responses	MSF	minutes	3.58	5.31	5.28	1.34	1.63	8.75
	MSI	minutes	7.08	9.82	9.82	1.29	1.71	11.67
	MSR	minutes	6.5	8.52	8.51	1.02	0.96	10.5
Red blood cells	HT	%	27	31.75	31.50	2.79	3.5	38
	HB	(g/dL^−1^)	8	9.59	9.85	0.89	1.18	11.1
	RBC	(×10^6^/mL)	9.43	11.68	11.77	1.15	1.75	13.56
	MCV	pg/hm	23.6	27.16	26.95	2.19	1.75	34.6
	MCH	fl	7.1	8.15	8.17	0.38	0.43	8.6
White blood cells	LYM	cells/mL^−1^	1890.00	3491.60	3235.50	1004.70	1703.75	5427.00
	MONO	cells/mL^−1^	49	140.22	120.00	90.91	73	414
	EOS	cells/mL^−1^	58	333.42	267.50	287.51	125.57	1330.00
	LEC	cells/mL^−1^	11	6715.55	6900.00	2057.05	1425.00	10,300.00
	SEG	cells/mL^−1^	980	3282.35	3200.000	1083.10	1248.75	5280.00
Performance	CCW	kg	6	9.98	9.55	2.55	3.63	14.6
	HCW	kg	6.8	10.66	10.30	2.56	3.9	15.2
	FCW	kg	19	26.32	26.00	4.44	5.25	34
Non-carcass components	BLOOD	L	0.65	0.95	0.89	0.21	0.3	1.48
	LUNG	kg	0.37	0.5	0.48	0.08	0.14	0.63
	HEART	kg	0.05	0.09	0.09	0.02	0.02	0.13
	KIDNEYS	kg	0.06	0.08	0.08	0.01	0.02	0.11
	LIVER	kg	0.27	0.4	0.4	0.07	0.1	0.56
	SPLEEN	kg	0.01	0.06	0.04	0.07	0.02	0.34
Commercial meat cuts	SHANK	kg	1.05	1.62	1.55	0.35	0.5	2.27
	RIB	kg	0.26	0.74	0.72	0.26	0.36	1.21
	LOIN	kg	0.27	0.47	0.46	0.16	0.26	0.8
Morphological responses	TP	cm	52	60.05	60.250.	4.53	5.88	67
	BL	cm	44	50.93	50.75	4.1	5.5	59
	SHANKCIRC	cm	22	24.25	24.00	1.63	2.5	28
	LEA	cm	12.42	21.8	20.17	5.67	5.37	36.1
Meat traits	FMARB	1–5	1	1.6	2.0	0.5	1	2
	FTEXT		2	2.9	3.0	0.31	0	3
	FTHICK		0.12	1.83	1.77	1.05	1.25	3.9
	MTEXT		3	4.05	4.0	0.6	0	5
Meat physical characteristics	IpH	pH	4.25	5.2	5.28	0.54	0.67	6.21
	FpH	pH	4.45	5.98	6.00	1.28	1.45	9.95
	IT	°C	26.8	29.85	29.75	1.88	2.73	32.9
	FT	°C	10.6	13.15	13.00	1.26	2	15

Min: minimum; SD: standard deviation; IQR: interquartile range; Max: maximum. RR: respiratory rate; HR: heart rate; RT: rectal temperature; MSF: minutes spent feeding; MSI: minutes spent in idleness; MSR: minutes spent ruminating; HT: hematocrit; HB: hemoglobin; RBC: red blood cells; MCV: mean corpuscular volume; MCH: mean corpuscular hemoglobin; white blood cells—LYM: lymphocyte; MONO: monocyte; EOS: eosinophile; LEC: leukocytes; SEG: segmented cells; CCW: cold carcass weight; FCW: fasting carcass weight; HCW: hot carcass weight; BLOOD: blood volume; LUNG: lung weight; HEART: heart weight; KIDNEYS: kidney weight; LIVER: liver weight; SPLEEN: spleen weight; SHANK: shank weight; RIB: rib weight; LOIN: loin weight; TP: thorax perimeter; BL: body length; SHANKCIR: shank circumference; LEA: loin eye area; FMARB: fat marbling; FTEXT: fat texture; FTHICK: fat thickness; MTEXT: meat texture; IpH: initial pH; FpH: final pH; IT: initial temperature; FT: final temperature.

**Table 3:** Canonical correlation metrics.

Dependent Variables	Independent Variables	Canonical R	Canonical R^2^	*p*-Value
		U_1_. V_1_	U_1_. V_1_	
RR, HR, RT	MSF, MSR, MSI	0.67	0.45	=0.07
RR, HR, RT	HT, HB, RBC, MCV, MCH	0.73	0.53	=0.52
RR, HR, RT	LYM, MONO, EOS, LEC, SEG	0.66	0.44	=0.67
RR, HR, RT	CCW, HCW, FCW	0.81	0.66	=0.03
RR, HR, RT	TP, BL, SHANKCIRC, LEA	0.82	0.68	=0.03
RR, HR, RT	BLOOD, LUNG, HEART, KIDNEYS, LIVER, SPLEEN	0.91	0.82	<0.001
RR, HR, RT	SHANK, RIB, LOIN	0.82	0.67	=0.03
RR, HR, RT	FMARB, FTEXT, MTEXT, FTHICK	0.58	0.34	=0.44
RR, HR, RT	IpH, FpH, IT, FT	0.49	0.24	=0.92

*p*-value: *p*-value obtained in the application of the Wilks’ lambda significance test. RR: respiratory rate; HR: heart rate; RT: rectal temperature; MSF: minutes spent feeding; MSI: minutes spent in idleness; MSR: minutes spent ruminating; HT: hematocrit; HB: hemoglobin; RBC: red blood cells; MCV: mean corpuscular volume; MCH: mean corpuscular hemoglobin; white blood cells—LYM: lymphocyte; MONO: monocyte; EOS: eosinophile; LEC: leukocytes; SEG: segmented cells; CCW: cold carcass weight; FCW: fasting carcass weight; HCW: hot carcass weight; BLOOD: blood volume; LUNG: lung weight; HEART: heart weight; KIDNEYS: kidney weight; LIVER: liver weight; SPLEEN: spleen weight; SHANK: shank weight; RIB: rib weight; LOIN: loin weight; TP: thorax perimeter; BL: body length; SHANKCIR: shank circumference; LEA: loin eye area; FMARB: fat marbling; FTEXT: fat texture; FTHICK: fat thickness; MTEXT: meat texture; IpH: initial pH; FpH: final pH; IT: initial temperature; FT: final temperature.

**Table 4:** MANOVA summary for the main indicators that influence the thermoregulatory responses of sheep.

Indicator	Variable	Df	Pillai	Approx F	Num Df	Den Df	Pr (>F)
Behavioral responses	MSF	1.00	0.23	1.41	3.00	14.00	0.28
	MSR	1.00	0.18	1.00	3.00	14.00	0.42
	MSI	1.00	0.43	3.54	3.00	14.00	0.04
	Residuals	16.00					
Performance	CCW	1.00	0.59	6.80	3.00	14.00	<0.001
	HCW	1.00	0.29	1.93	3.00	14.00	0.17
	FCW	1.00	0.19	1.07	3.00	14.00	0.39
	Residuals	16.00					
Morphological responses	TP	1.00	0.56	5.53	3.00	13.00	0.01
	BL	1.00	0.53	4.83	3.00	13.00	0.02
	SHANKCIRC	1.00	0.17	0.92	3.00	13.00	0.46
	LEA	1.00	0.01	0.05	3.00	13.00	0.98
	Residuals	15.00					
Non-carcass components	Blood	1.00	0.68	7.88	3.00	11.00	<0.001
	Lung	1.00	0.46	3.13	3.00	11.00	0.07
	Heart	1.00	0.27	1.33	3.00	11.00	0.31
	Kidneys	1.00	0.11	0.43	3.00	11.00	0.73
	Liver	1.00	0.59	5.26	3.00	11.00	0.02
	Spleen	1.00	0.59	5.32	3.00	11.00	0.02
	Residuals	13.00					
Carcass commercial cuts	Shank	1.00	0.60	7.02	3.00	14.00	<0.001
	Rib	1.00	0.34	2.43	3.00	14.00	0.11
	Loin	1.00	0.07	0.36	3.00	14.00	0.78
	Residuals	16.00					

SF: minutes spent feeding; MSI: minutes spent in idleness; MSR: minutes spent ruminating; CCW: cold carcass weight; FCW: fasting carcass weight; HCW: hot carcass weight; TP: thorax perimeter; BL: body length; SHANKCIR: shank circumference; LEA: loin eye area; BLOOD: blood volume; LUNG: lung weight; HEART: heart weight; KIDNEYS: kidney weight; LIVER: liver weight; SPLEEN: spleen weight; SHANK: shank weight; RIB: rib weight; LOIN: loin weight.

## Data Availability

Data will be made available on request.

## Abbreviations

BL, body length; BLOOD, blood volume; CCW, cold carcass weight; EOS, eosinophile; FCW, fasting carcass weight; FMARB, fat marbling; FpH, final pH; FT, final temperature; FTEXT, fat texture; FTHICK, fat thickness; HB, hemoglobin; HCW, hot carcass weight; HEART, heart weight; HR, heart rate; HT, hematocrit; IpH, initial pH; IT, initial temperature; KIDNEYS, kidney weight; LEA, loin eye area; LEC, leukocytes; LIVER, liver weight; LOIN, loin weight; LUNG, lung weight; LYM, lymphocyte; MCH, mean corpuscular hemoglobin; MCV, mean corpuscular volume; MONO, monocyte; MSF, minutes spent feeding; MSI, minutes spent in idleness; MSR, minutes spent ruminating; MTEXT, meat texture; RBC, red blood cells; RIB, rib weight; RR, respiration rate; RT, rectal temperature; SEG, segmented cells; SHANK, shank weight; SHANKCIRC, shank circumference; SPLEEN, spleen weight; TP, thorax perimeter.
